# Occult hepatitis B infection in children born to HBeAg-positive women confers a low long-term risk for HBsAg-positive infection

**DOI:** 10.1007/s15010-024-02290-1

**Published:** 2024-05-09

**Authors:** Anders Eilard, Maria E. Andersson, Rune Wejstål, Gunnar Norkrans, Magnus Lindh

**Affiliations:** 1 Department of Infectious Diseases, University of Gothenburg, 413 46 Gothenburg, Sweden; 2 Department of Infectious Diseases, Sahlgrenska University Hospital, 416 85 Gothenburg, Sweden

**Keywords:** Occult, OBI, Hepatitis B virus, Mother-to-child

## Abstract

**Purpose:**

Mother-to-child transmission (MTCT) has been the main cause of chronic hepatitis B virus (HBV) infection, particularly in East Asia. Hepatitis B immunoglobulin (HBIG) and vaccination given directly after birth effectively prevents hepatitis B surface antigen (HBsAg)-positive (overt) HBV infection, but occult hepatitis B infection (OBI) may develop despite adequate prophylaxis. The aim of this study was to investigate the long-term outcome in children born to mothers with very high HBV DNA levels with special focus on children discovered in early childhood with OBI.

**Methods:**

One-year and long-term outcome regarding overt and occult HBV infection were analysed in 66 children born to hepatitis B e antigen (HBeAg)-positive mothers, and were compared with one-year outcome in 69 children born to HBeAg-negative mothers. The children were born between 1998 and 2018.

**Results:**

Six children born to HBeAg-positive mothers developed overt chronic HBV infection, in two cases after normal pregnancies and despite HBIG and vaccination, but never when nucleotide analogue treatment was given during pregnancy. OBI with HBV DNA detected in serum in the absence of surface antigen (HBsAg) was observed in four children at the age of 1 year. One of them was transiently HBsAg-positive at the age of 7 years. At long-term follow-up, six children had overt chronic infection, one had OBI and six had previous OBI or positive anti-HBc suggesting resolved unidentified infections.

**Conclusion:**

The results indicate that children born to mothers with high HBV DNA levels have approximately 10% risk to develop OBI despite antiviral treatment, vaccination and HBIG, but that such OBI confers a minimal long-term risk for overt infection, at least in immunocompetent children.

## Introduction

Chronic hepatitis B virus (HBV) infection affects more than 250 million individuals worldwide and is a major cause of liver cirrhosis and hepatocellular carcinoma (HCC) [1]. Vertical transmission from mother-to-child during delivery (MTCT) has been the main cause of chronic HBV infection in high prevalence areas such as East Asia [2]. Early post-natal administration of vaccine and HBV specific immunoglobulins (HBIG), and complementary antiviral treatment of selected pregnant women, can drastically reduce transmission of overt (HBsAg-positive) infection and long-term complications [3].

The effect of these preventive measures on occult HBV infection (OBI) is less well known. OBI is defined as a condition where low levels of HBV DNA can be found in the blood or in liver tissue although hepatitis B surface antigen (HBsAg) is not detected in serum [4]. It is well known that low HBV DNA levels can remain after loss of HBsAg following acute or chronic HBV infection, a phenomenon called secondary OBI. Primary OBI is a rarely recognized condition with detection of low levels of HBV DNA in a person who has not experienced an overt HBV infection. A dose dependent risk for primary OBI upon exposure to a low infectious dose has been demonstrated in the woodchuck model [5]. Primary OBI has also been described in children born to women with HBV infection, including when vaccine and HBIG have been administered [6–8]. In a previous study we identified primary OBI at the age of 1 year in three out of eight children born to hepatitis B e-antigen (HBeAg) positive women, and in one of the them OBI was still present at the age of 5 years, whereas in another child overt HBV infection with low levels of HBV DNA and HBsAg had developed at the age of 7 years [9]. These findings encouraged us to investigate potential long-term effects of primary OBI further. Therefore, in the present study we included children born to HBeAg-positive and HBeAg-negative women between 1998-2018 with the aim of investigating the prevalence of primary OBI in a larger cohort and to determine the long-term outcome after different prophylactic strategies.

## Subjects and methods

### Overt and occult HBV infection

Overt HBV infection is defined as an HBsAg-positive chronic HBV infection with or without detectable HBV DNA in serum.

Occult HBV infection (OBI) is defined as an HBsAg-negative chronic HBV infection with presence of replication-competent HBV DNA (covalently closed circular DNA, cccDNA) in the liver and detectable or undetectable HBV DNA in serum [4]. In this study, where no liver biopsies were performed, detection of HBV DNA in serum was a requirement for an OBI classification. Also, a positive anti-HBc together with HBsAg-negativity was interpreted as a probable OBI (seropositive OBI)[4].

### Study population

HBsAg screening is included in the standard of care for all pregnant women in Sweden. Those testing positive are referred to a tertiary centre for prophylactic recommendations. All HBsAg- and HBeAg-positive pregnant women referred to the Infectious Diseases Department at the Sahlgrenska University Hospital in Gothenburg, Sweden, during the period 2003–2016 were invited to participate in the present study together with their child. Siblings of that child were also invited to participate, provided that their mothers were still HBeAg-positive during their pregnancies. In total, 52 HBeAg-positive women with 91 children were identified and 37 women together with 66 children born between 1998 and 2018 agreed to participate and were included. Six of the 66 children had participated in a previous study [9], including three that were OBI-positive at the age of 1 year. An age matched comparison group (n = 69) was randomly selected from children born to HBsAg-positive, HBeAg-negative pregnant women that had been referred to the same department during the study period.

### HBV prophylaxis

With a few exceptions the children had been given immunoprophylaxis against hepatitis B according to the general Swedish recommendations. Thus, all children had received hepatitis B vaccine immediately after birth (Engerix-B, 10 μg HBsAg, GlaxoSmithKline), and children born to HBeAg-positive mothers had also simultaneously received HBIG (Umanbig, 180 IU, Scandinavian Biopharma). HBIG was not administered to children born to HBeAg-negative mothers unless individually decided.

Subsequent HBV vaccine doses were given according to guidelines. During 1998–2016 three additional Engerix-B doses were given at 2, 6 and 52 weeks of age and HBV serology (HBsAg, anti-HBc, anti-HBs) were tested at the age of 52 weeks. From 2016, another Engerix-B dose was given at 1 month of age, followed by three doses as part of the combination vaccine in the general vaccination program (Infanrix® hexa, GlaxoSmithKline) at 3, 5 and 12 months of age. HBV serology tests were performed at the age of 18 months.

From 2009–2016 all HBsAg-positive pregnant women with HBV DNA ≥ 7.5 log_10_ IU/mL were offered antiviral treatment with a nucleotide analogue from gestation week 32 until delivery and in 2016 the threshold for treatment was lowered to HBV DNA ≥ 5.3 log_10_ IU/mL.

All children were examined for HBsAg, anti-HBs, anti-HBc IgG and HBV DNA at the age of 12–18 months. Children born to HBeAg-positive women were evaluated with a new serum sample taken at the time of inclusion in the present study. All samples for long-term follow up in this group were collected between 2020–2022.

### HBV serology and HBV DNA

HBsAg, anti-HBc IgG and anti-HBs were analysed by Abbott AxSym (1996–2000), Abbott Architect (2001–2018) or Abbott Alinity (2018–2022) (Abbot Diagnostics, Chicago, Ill.). HBV DNA quantification was performed with the AmpliPrep/Taqman HBV Test v2.0 or Cobas 6800 assays (Roche Diagnostics, Branchburg, NJ) which has a lower quantification limit at 1.0 log IU/mL. Results in copies/mL (prior to a shift to IU/mL in 2005) were translated to IU/mL by dividing by 5.82 (corresponds to subtraction by 0.76 log_10_ units). Genotyping was performed by real-time PCR [10] or by sequencing and phylogeny of a partial segment of the S region (nt 593–1022).

## Results

**Fig. 1:**
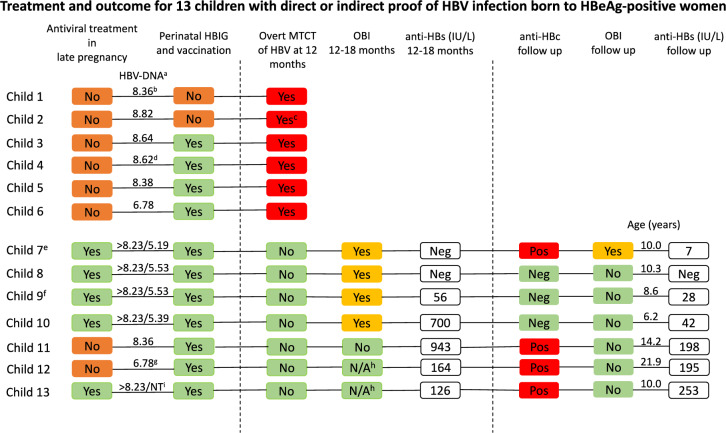
Treatment and outcome for 13 children with direct or indirect proof of HBV infection born to HBeAg-positive women ^a^ HBV DNA in log_10_ IU/mL. Prenatal values with addition of level at delivery for women with antiviral treatment. ^b^ HBV DNA tested 1.5 years postpartum. ^c^ Child 2 was tested at the age of 3.4 years. ^d^ HBV DNA tested 4.8 years postpartum. ^e^ Child 7 had a period of evident overt infection at the age of 7 years. ^f^ Child 9 also had OBI at 5 years of age. ^g^ HBV DNA tested 1.8 years postpartum. ^h^ Not Available. Stored sera for children 12 and 13 were not sufficient to perform HBV DNA at long term follow up. ^i^ Not Tested

Table 1 shows the baseline characteristics for 37 HBeAg-positive and 68 HBeAg-negative women together with their children. In the HBeAg-positive group 66 children were born in separate pregnancies with a mean of 1.8 (range 1- 4) children/woman. The HBV genotype reflected the geographic origin of the mothers: The 29 with genotype B, C or I had East Asian origin, the 7 with genotype D originated from South Europe, Middle East or North Africa, and one with genotype E from West Africa. In the HBeAg-negative comparison group, 69 children were born in separate pregnancies. Treatment and outcome for the children with documented infection with HBV are presented in Fig. 1 and Table 2.

**Table 1:** Baseline characteristics of mothers and children

	HBeAg-positive	HBeAg-negative
*Mothers*	n = 37	n = 68
Age (years)^a^	28.7 ± 5.4	32.1 ± 5.4
ALT/ULN^a,b^	0.45 ± 0.23	0.43 ± 0.22
Antenatal HBV DNA (log_10_ IU/mL)^c^	8.04 (2.71 – > 9.68)	2.86 (< 1 – 7.86)
HBV genotype	13 B, 15 C, 7 D, 1 E, 1 I	ND^d^
*Children*	n = 66	n = 69
Gestational age, weeks^c^	39.0 (24.6 – 42.0)	40.3 (33.4 – 42.1)
Caesarean section	14.8%	12.3%
Height, cm^c^	50 (33 – 53)	50 (39–55)
Weight, kg^c^	3.25 (0.71 – 4.65)	3.5 (2.4 – 4.4)
Apgar score (1 min)^c^	9 (2 – 10)	9 (3 – 10)
Age at follow up, years (mean)	12.3	12.6

Genotype was not recorded for the HBeAg negative mothers

^a^ Mean ± standard deviation

^b^ Upper Limit of Normal

^c^ Median (range)

^d^ Not Determined

**Table 2:** Baseline characteristics and outcome in three different groups, HBeAg-positive women with or without antiviral treatment in late pregnancy and HBeAg-negative women with their corresponding children (vaccination only)

	HBeAg-positive antiviral treatment^a^	HBeAg-positive no antiviral treatment^a^	HBeAg-negative^a^
*Mothers*	n = 17	n = 28	n = 68
Age (years)	29.2	28.5	32.0
Antenatal HBV DNA^b^ (log_10_ IU/ml)	8.23 (3.30 – 8.69)	8.04 (2.71 – > 9.68)	2.86 (< 1 – 7.86)
HBV DNA at birth^b^ (log_10_ IU/ml)	5.39 (< 1.0 – 7.35)	NT^c^	NT^c^
*Children*	n = 21	n = 45	n = 69
Gestational age (weeks)^b^	39.1 (33.4–41.8)	38.0 (24.6–42.0)	40.3 (33.4–42.1)
Apgar score (1 min)	9 (8–10)	9 (2–10)	9 (3–10)
Mean age at follow up (years)	7.4	14.7	12.6
Overt MTCT of HBV infection	0^d^	4^e^	0
OBI at age of 1 year	4	0	0
Anti-HBc positive at follow-up without prior OBI detected	1	2	NT^c^
Detectable HBV DNA at follow-up	1	0	NT^c^
Anti-HBs at age 12–18 months^b^ (IU/mL)	81 (0—> 1000)	176 (0—> 1000)	326 (0—> 1000)
Anti-HBs at follow up^b^ (IU/mL)	106 (0—> 1000)	43 (0—> 1000)	NT^c^

^a^ All children to HBeAg-positive women received vaccination + HBIG and children to HbeAg-negative women received vaccination only

^b^ Median (range)

^c^ Not Tested

^d^ Excluding one child being OBI-positive at the age of 1 years that later developed a short lasting, low-level overt infection

^e^ Excluding two children that did not receive any prophylaxis at all

### Overt infection after MTCT

Overt mother-to-child-transmission (MTCT) with detection of HBsAg at 1 year of age was found in six children. Two of the children did not receive any prophylaxis at all. The mother to child 1 (Fig. 1) was by mistake not screened for HBsAg at the maternity care centre in early pregnancy and the mother to child 2 left the country in late pregnancy and was temporarily lost to follow-up. Child 3 was small for gestational age at birth but received accurate vaccination and HBIG. Child 4 was born after a normal pregnancy and received full prophylaxis according to the schedule. Child 5 was born by emergency caesarean section and immediately received postnatal vaccination and HBIG. All mothers to children 1–5 had very high HBV DNA levels (above 8.0 log_10_ IU/mL) during pregnancy or in samples taken 1–5 years later. The five children developed HBeAg-positive infection, and four of them still had HBV DNA levels above 8 log_10_ IU/mL at 20 years of age. Child 6 was born prematurely and received accurate postnatal vaccination and HBIG, but had the second vaccine dose late, at the age of 7 months. The mother had a high HBV DNA level (6.78 log_10_ IU/mL) during pregnancy and the child became HBeAg-negative with HBV DNA below 3.0 log_10_ IU/mL from 2 years of age. None of the 21 children whose 17 mothers had antiviral treatment during the last trimester of the pregnancy developed overt HBV infection.

Among children born to HBeAg-positive mothers the risk for having overt HBV infection at 1 year of age was 9% (4/43) in the group with adequate postnatal prophylaxis but no antiviral treatment during the last trimester, and 0% (0/22) in the group with adequate postnatal prophylaxis and antiviral treatment of the mother.

### OBI

Stored serum samples taken at the age of 1 year were available for 40 of the 66 children that were born to HBeAg-positive mothers. Primary OBI (detection of HBV DNA in serum) at the age of 1 year was identified in four of these children, and in no child in the HBeAg-negative group (n = 69). The four children with OBI were born to mothers that received antiviral treatment in the last trimester of pregnancy and had received vaccine and HBIG immediately postpartum and completed the vaccination series. All four children had HBV DNA levels below 200 IU/mL and 3 out of 4 had high enough HBV DNA levels to perform sequencing, which showed close correlation with the sequence of their respective mother [9].

Child 7 with OBI at the age of 1 year, and overt HBV infection (HBsAg-positive) at the age of 5 years, again had OBI at the age of 10.0 years with positive HBV DNA below the quantification limit, anti-HBs 7 IU/L and positive anti-HBc. Children 8–10 with OBI at the age of 1 year were negative for both HBV DNA and anti-HBc at follow-up (at ages 6.2, 8.6 and 10.3 years). Children 11–13 without OBI at 1 year of age were anti-HBc-positive at follow-up (at ages 10.0, 14.2 and 21.9 years). Another three children had borderline positive anti-HBc at follow up which were classified as false positive. Thus, at follow-up (at a median age of 12.3 years), one child in the HBeAg-positive group had OBI, 3 had previous OBI and 3 had positive anti-HBc indicating previous exposure to HBV.

### Anti-HBs antibodies

The anti-HBs levels at 1 year of age, reflecting response to HBV vaccination, were higher in the group of children that were born to HBeAg-negative compared with those born to HBeAg-positive mothers. Additionally, the four children in the HBeAg-positive group, who were anti-HBc positive at follow-up, had significantly higher anti-HBs levels at the late follow-up compared with the other children in the same cohort.

## Discussion

The present study investigated the risk for children born to women with chronic HBV infection to develop OBI or overt HBV infection. Six out of 66 children with HBeAg-positive mothers developed overt chronic HBV infection, including four with breakthrough infections and two that had not received prophylaxis. Of the remaining 60 children, one still had OBI at follow-up at 10 years of age, that is, was HBV DNA-positive and HBsAg-negative. That child had OBI at the age of 1 year, followed by a period with overt infection (HBsAg-positive) at the age of 7 years. The other 59 children were HBsAg- and HBV DNA-negative in serum at long-term follow-up. Three of these 59 had presented with transient OBI at the age of 1 year. Out of four children with OBI at 1 year of age, only the child with overt infection at the age of 7 years developed anti-HBc antibodies. However, three additional children (with no identified OBI) were anti-HBc-positive at follow-up. Taken together, the long-term follow-up showed that that out of 66 children born to HBeAg-positive mothers, 6 children had become chronically infected, one had OBI and 6 had findings indicating a previous infection (OBI or isolated anti-HBc).

Four of the children that developed overt chronic HBV infection had received vaccine and HBIG directly after birth, but their mothers had not received antiviral treatment during pregnancy despite high HBV DNA levels, since they were born prior to 2009 when this practice was introduced. Previous studies have shown that treating mothers with high HBV DNA levels with antivirals in late pregnancy significantly reduces the risk for overt HBV infection by MTCT [11–14]. The present study was initiated by our earlier finding that OBI was relatively frequent also if antiviral treatment was prescribed in late pregnancy [9], and we had expected to find more cases of OBI among the children born to HBeAg-positive mothers than we did. One explanation might be that MTCT had resulted in overt chronic infection rather than in OBI when antiviral prophylactic treatment was not applied, another that some cases of OBI at 1 year of age were not detected because of the longer storage time of the samples as compared with the former study, which is likely to be of importance if the HBV DNA level is very low as is the case in OBI.

While strategies to prevent overt HBV infection in children born to HBsAg-positive mothers have developed over time, including antiviral treatment during pregnancy [15, 16], the knowledge on how to prevent OBI has remained poor. There has even been lack of knowledge about the frequency of OBI in children born to HBsAg-positive mothers with OBI frequencies ranging from 0% [17–19] to 44% [20] in a recent meta-analysis [21]. To some extent this may reflect differences in the definition of OBI-cases. In studies disclosing HBV DNA levels [6, 22–29], 40% (range 7% [25] – 100% [22, 23, 27–29]) of the OBI-cases had HBV DNA > 1200 copies/mL, which is not in line with the established criteria for OBI stating that HBV DNA should be below 200 IU/mL (≈ 1200 copies/mL) [30]. A Danish study [19] found no cases of OBI but that 17% were anti-HBc-positive at an age between 1.9–6.4 years. A Taiwanese study [8] on children with HBeAg-positive mothers observed no impact on OBI by antiviral treatment in late pregnancy (approximately 3.5% in children born to both treated and untreated mothers measured at the age of 12 months), possibly reflecting transplacental transmission prior to initiation of antiviral treatment. Interestingly, that study identified one child with OBI developing into overt infection, like our observation. Further, that study analysed samples taken at both 6 and 12 months of age and found that 13 of the 14 cases with OBI at 6 months had disappeared at 12 months, when 7 new cases were detected. This could be an indication that OBI may appear at different ages and be short-lasting, causing an underestimation of the prevalence of MTCT in children that do not develop anti-HBc. However, one child in the present study was positive for OBI both at 1 and 5 years of age.

Most previous studies have reported OBI in 1–5% of children born to HBsAg-positive mothers, but it has not been established that viral load or HBeAg-status in the mother is associated with OBI in the children. Our data suggest that these factors play a role, because we found MTCT of HBV infection only among the 66 children born to HBeAg-positive mothers (including 6% with OBI) and not (neither OBI nor overt infection) in any of the 69 children born to HBeAg-negative mothers. The latter observation supports that administration of HBV vaccine alone directly after birth, without HBIG, provides adequate protection for children born to HBeAg-negative mothers with low viral load (HBV DNA < 200 000 IU/mL), in agreement with published data [31–33]. Based on the established low risk for children born to HBeAg-negative mothers we did not collect blood for late follow-up analyses of HBV DNA and anti-HBc in this group.

## Conclusion

In summary, we found that primary OBI after MTCT of HBV was relatively common in children born to HBeAg-positive women despite adequate prophylaxis but was not observed in any of the children born to HBeAg-negative women. Overt chronic infection was also relatively common in children with HBeAg-positive mothers born prior to the introduction of antiviral treatment in late pregnancy. Our study suggests that the introduction of antiviral treatment to mothers with very high HBV DNA levels may have caused a shift from overt HBV infection to OBI. At long-term follow-up only one case with OBI was HBV DNA positive, indicating that OBI is not a significant clinical problem. However, reactivation of an undetected OBI in case of immunosuppressive treatment later in life should be kept in mind as a potential explanation to apparently cryptogenic HBV infection.

## Data Availability

Data is available upon request to the corresponding author.
